# Phenological responses of alpine snowbed communities to advancing snowmelt

**DOI:** 10.1002/ece3.11714

**Published:** 2024-07-14

**Authors:** Harald Crepaz, Elena Quaglia, Giampiero Lombardi, Michele Lonati, Mattia Rossi, Simone Ravetto Enri, Stefan Dullinger, Ulrike Tappeiner, Georg Niedrist

**Affiliations:** ^1^ Institute for Alpine Environment Eurac Research Bozen Italy; ^2^ Department of Ecology University of Innsbruck Innsbruck Austria; ^3^ Independent Researcher Torino Italy; ^4^ Department of Agricultural, Forest and Food Sciences, University of Torino Università degli Studi di Torino Grugliasco Italy; ^5^ NBFC, National Biodiversity Future Center Palermo Italy; ^6^ European Commission Ispra Italy; ^7^ Institute for Earth Observation Eurac Research Bozen Italy; ^8^ Department of Botany and Biodiversity Research University of Vienna Vienna Austria

**Keywords:** alpine ecosystem, phenocam, phenophase, predictive modelling, snow cover

## Abstract

Climate change is leading to advanced snowmelt date in alpine regions. Consequently, alpine plant species and ecosystems experience substantial changes due to prolonged phenological seasons, while the responses, mechanisms and implications remain widely unclear. In this 3‐year study, we investigated the effects of advancing snowmelt on the phenology of alpine snowbed species. We related microclimatic drivers to species and ecosystem phenology using *in situ* monitoring and phenocams. We further used predictive modelling to determine whether early snowmelt sites could be used as sentinels for future conditions. Temperature during the snow‐free period primarily influenced flowering phenology, followed by snowmelt timing. *Salix herbacea* and *Gnaphalium supinum* showed the most opportunistic phenology, while annual *Euphrasia minima* struggled to complete its phenology in short growing seasons. Phenological responses varied more between years than sites, indicating potential local long‐term adaptations and suggesting these species' potential to track future earlier melting dates. Phenocams captured ecosystem‐level phenology (start, peak and end of phenological season) but failed to explain species‐level variance. Our findings highlight species‐specific responses to advancing snowmelt, with snowbed species responding highly opportunistically to changes in snowmelt timings while following species‐specific developmental programs. While species from surrounding grasslands may benefit from extended growing seasons, snowbed species may become outcompeted due to internal‐clock‐driven, non‐opportunistic senescence, despite displaying a high level of phenological plasticity.

## Graphical abstract

Our study investigates the phenological responses of five typical snowbed species to advancing snowmelt using *in situ* monitoring, phenocam imagery and predictive modelling. Over 3 years, we found temperature to be the primary driver of phenological changes in alpine snowbeds, with varying importance of photoperiodism among species. Results suggest a potential diminishing relevance of photoperiodism in warmer conditions.

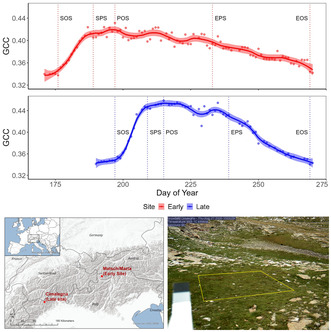

## 1 INTRODUCTION

Alpine grasslands, characterised by a heterogeneous topographic and microclimatic mosaic (Scherrer & Körner, 2011), are undergoing significant transformation due to global warming, which disproportionately impacts plant growth in high‐latitude and alpine regions (Vorkauf, Kahmen, et al., 2021). This transformation is driven primarily by rising temperatures and declining snow cover (Klein et al., 2016; Matiu et al., 2021). Even if snowfall at higher elevations is not expected to decline as much as that at lower elevations (Serquet et al., 2011; Vorkauf, Marty, et al., 2021), increasing temperatures and thus lower amounts of snow during the spring months will result in reduced snow cover, earlier melt‐out dates and prolongation of the phenological season (Körner et al., 2023; Möhl et al., 2022; Vorkauf, Kahmen, et al., 2021). Future climate scenarios predict the continuation of this trend in the 21st century (Calvin et al., 2023; Hock et al., 2019). As a result, cold‐ and snow‐dependent biota, such as alpine snowbed communities, are among the most vulnerable habitats to climate change (Carbognani et al., 2014; Kivinen et al., 2012; Matteodo et al., 2016). However, the impacts of warming on these communities need to be further investigated, as snowbed species may be less affected than other alpine species due to their snow cover's greater dependence on topography and wind patterns than snowfall (Körner & Hiltbrunner, 2021).

Snowbeds are habitats form in small topographical depressions in the alpine zone (Björk & Molau, 2007) where high amounts of snow accumulate during winter, resulting in late melt‐out, thereby protecting the vegetation from harsh winds and temperatures below the freezing point. Ecosystems developing at these sites are characterised by low plant biomass yields, high disturbance rates and free space available for colonisation (Jabis et al., 2020; Vittoz et al., 2009). As freeze–thaw cycles are rare, plant species adapted to snowbeds are particularly vulnerable to spring frosts, unlike their more generalist counterparts on adjacent ridges (Carbognani et al., 2012; Petraglia et al., 2014). These are often more competitive and might replace snowbed specialists as soon as snowmelt allows for their successful local establishment (Choler et al., 2001; Heegaard & Vandvik, 2004; Lyu & Alexander, 2022). Therefore, changes in snow cover may substantially affect the diversity, composition and functionality of snowbed communities (Jabis et al., 2020; Vittoz et al., 2009). Observational evidence already revealed more pronounced alterations occurring in high alpine plant communities compared to other ecosystems (Carbognani et al., 2012; Matteodo et al., 2013; Pauli et al., 2012; Steinbauer et al., 2018). These impacts have already demonstrated the potential to shift the distribution ranges of alpine species and restructure entire community assemblages (Carbognani et al., 2014; Crepaz et al., 2021; Rixen et al., 2010). The drivers of these ecological changes in alpine ecosystems are increasing temperatures (Bjorkman et al., 2020; Kopp & Cleland, 2015) and advancing snowmelt (Iler et al., 2013; Petraglia et al., 2014; Wipf et al., 2009), as snowpack depth, duration and melt out control the onset and length of the phenological season in cold regions (Alecrim et al., 2023; Freppaz et al., 2008; Semenchuk, 2013). Onset and length of the phenological season also control phenological timing, which is crucial for the seasonal progression of plant activity through dormancy, growth, reproduction and senescence. Consequently, advancing snowmelt has already been shown to alter the phenological cycles of alpine plant species (Abeli et al., 2012; Barrett & Hollister, 2016; Bjorkman et al., 2015). Alpine plants have developed various strategies to cope with the sudden release from snow and prevent substantial, persistent shifts in phenology that could disrupt biological interactions, such as pollination or trophic relationships (Leingärtner et al., 2014; Li et al., 2022). These strategies include remaining green and retaining full photosynthetic capacity over winter, flushing conventionally after snowmelt, surviving as seeds and germinating after (Galen & Stanton, 1995; Körner, 2021). However, even within a single snowbed habitat, species exhibit different developmental dynamics and variable phenological responses to varying snow conditions (Körner, 2021). Oberbauer et al. (2013) identified several alpine plant species whose phenology did not directly respond to temperature but was likely modulated through the regulatory role of daylength (photoperiodism). This mechanism is assumed to prevent plants from initiating vegetative development too early or too late in the phenological season (Körner, 2023), which could lead to freezing damage (Klein et al., 2016), reduced plant growth, or reduced reproductive potential for the entire vegetative cycle (Gezon et al., 2016; Inouye, 2008). Similar results were reported by Vorkauf, Kahmen, et al. (2021) in the Swiss Alps as they identified grassland species whose phenology tracked the snowmelt date, responding to temperature sums solely after the daylength indicated a low‐risk period for sub‐freezing temperatures. This strategy minimises freezing risk while conferring a competitive advantage to species primarily driven by temperature, as they can more opportunistically exploit the extended phenological season. Consequently, phenology serves as an effective indicator for assessing a species' potential to adapt to, or even benefit from, earlier snowmelt induced by climate change (Schmid et al., 2017). While certain mechanisms have already been well studied, an ongoing debate questions whether the predominantly species‐specific responses to advancing snowmelt either benefit or disadvantage certain species (Körner et al., 2022), prompt significant alterations in vegetation composition, or whether species will demonstrate long‐term adaptability.

Therefore, phenological studies at the single‐species level (Ding et al., 2016; Ernakovich et al., 2014; Khorsand Rosa et al., 2015) are increasingly complemented by multi‐species approaches which take advantage of technical developments in remote and proximate sensing (Stendardi et al., 2019). Digital imagery has gained increasing interest in ecology over the past years, not only via the use of satellite imagery, but also in the form of phenocams with the aim to track phenological developments in vegetation communities. Phenocams automatically and repeatedly acquire detailed images of the red, green, blue (RGB) and/or near‐infrared (NIR) bands, substantially contributing to the acquisition of data on seasonal changes in vegetation due to phenology. Therefore, data derived from phenocam images have been widely used to understand the relationships between canopy phenology and ecosystem processes and to study changes in leaf physiology that are associated with changes in leaf colour (Richardson, 2019).

Although phenocams have proven to be useful tools for assessing the phenology, health and productivity of a multitude of ecosystems (Brown et al., 2016; Richardson, 2019; Rossi et al., 2019), they have rarely been used to assess the phenology and productivity of alpine grasslands (Andreatta et al., 2023; Gallmann et al., 2022; Mann et al., 2022). Hence, it remains unclear whether phenocam imagery can contribute to our understanding of phenological development at the species level. Therefore, we aimed to determine whether the analysis of alpine grasslands using data derived from phenocams could provide a standardised and objective method for assessing phenological development at remote study sites. However, a disadvantage of these techniques is the limited information they provide at the species level. To maximise the ecological understanding, a combination of single‐species field data and multispecies digital imagery data may be optimal.

In this study, we therefore adopted an integrative approach combining phenological observations, modelling and the space‐for‐time approach to address the limitations of individual techniques (Piao et al., 2019) and enhance our understanding of future phenological responses to climate warming in alpine ecosystems (Niedrist et al., 2016; Pardee et al., 2019; Sherwood et al., 2017; Wipf et al., 2015). We utilised data from a late snowmelt site to represent current conditions and data from an early snowmelt site to serve as a proxy for a future climate change scenario with reduced snow cover and an extended phenological season. By comparing these contrasting site conditions, we aimed to generate reliable projections of phenological changes in alpine snowbed communities under advancing snowmelt driven by global warming. Our objectives were (1) to identify the primary drivers (temperature or snow cover) driving species' phenological development, (2) to evaluate the effectiveness of proximate sensing in understanding phenological responses to advancing snowmelt and depicting individual species development and (3) to use the space‐for‐time approach to gain insights into how snowbed species may respond to future climate change scenarios and whether arid alpine areas could serve as early indicators of future changes in phenological patterns.

## 2 MATERIALS AND METHODS

### 2.1 Study sites

**FIGURE 1:**
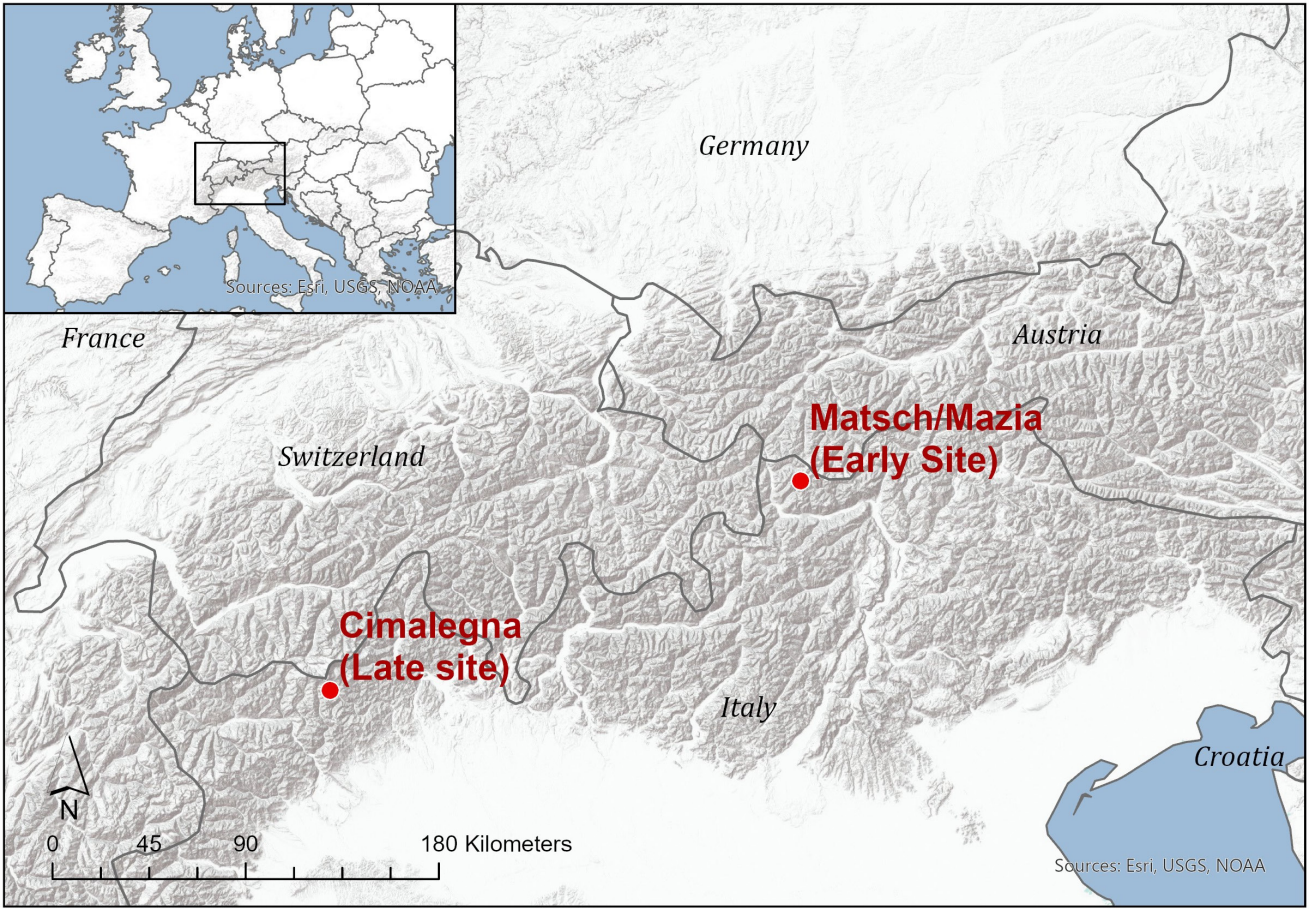
Topographical map of the Alps displaying the study sites: ‘Cimalegna’ (Late Snowmelt) and ‘Mazia’ (Early Snowmelt).

The study was conducted following a space‐for‐time approach at two long‐term ecological research (LTER) sites in the Italian Alps (Figure 1) over 3 years (2018–2020).

Study site 1, hereafter referred to as late site, is located on Cimalegna Plateau, an area close to Monte Rosa Massif (45°52′12.0″ N, 7°53′3.0″ E) at an elevation ranging between 2600 and 2900 m a.s.l. Site 2, hereafter referred to as early site, is located in Matsch/Mazia Valley (46°46′0.0″ N, 10°42′44.0″ E), at the same elevation. The vegetation communities at both study sites were mainly composed of two phytosociological associations: (a) *Salicetum herbaceae* in topographic depressions, forming the so‐called snowbed communities and (b) *Caricetum curvulae* on adjacent wind slopes and crests. While most site‐specific variables were very similar for both study sites, the cumulative average annual snowfall at the late Cimalegna site was 800 cm (Pintaldi et al., 2022), whereas the early Mazia site measured approximately 500 cm (https://browser.lter.eurac.edu). Consequently, the average snow cover duration was 260 days at the late snowmelt site (Cimalegna) and 235 days at the early snowmelt site (Mazia) (Table 1), resulting in an average difference of up to a month. During monitoring, the mean day of snowmelt (DOSM) difference between early and late sites was 12 days, markedly less than the 30 days reported by (Quaglia et al., 2020). Differences in snowmelt may be attributed to the measurements taking place under snowbed conditions (Körner et al., 2022). Moreover, an exceptionally heavy late May 2019 snowfall event notably delayed the start of snowmelt at the early site (Figure 1).

**TABLE 1:** Characteristics of the late and early snowmelt sites compared to day of snowmelt (DOSM), snow cover, elevation and vegetation.

Parameter	Late site	Early site
Avg. DOSM (days)	189 (178–194)	177 (173–181)
DOSM 2018 (days)	194	164
DOSM 2019 (days)	182	193
DOSM 2020 (days)	191	175
Snow cover (avg.)	260 days	235 days
Elevation (m a.s.l.)	2600–2900	
*T*_Soil_ (avg.) (°C)	9.8	10.4
Vegetation	*Salicetum herbaceae*	

*Note*: The DOSM is divided between average values, ranges and yearly values and indicated in days. Snow cover shows the average number of days with snow cover per year.

**FIGURE 2:**
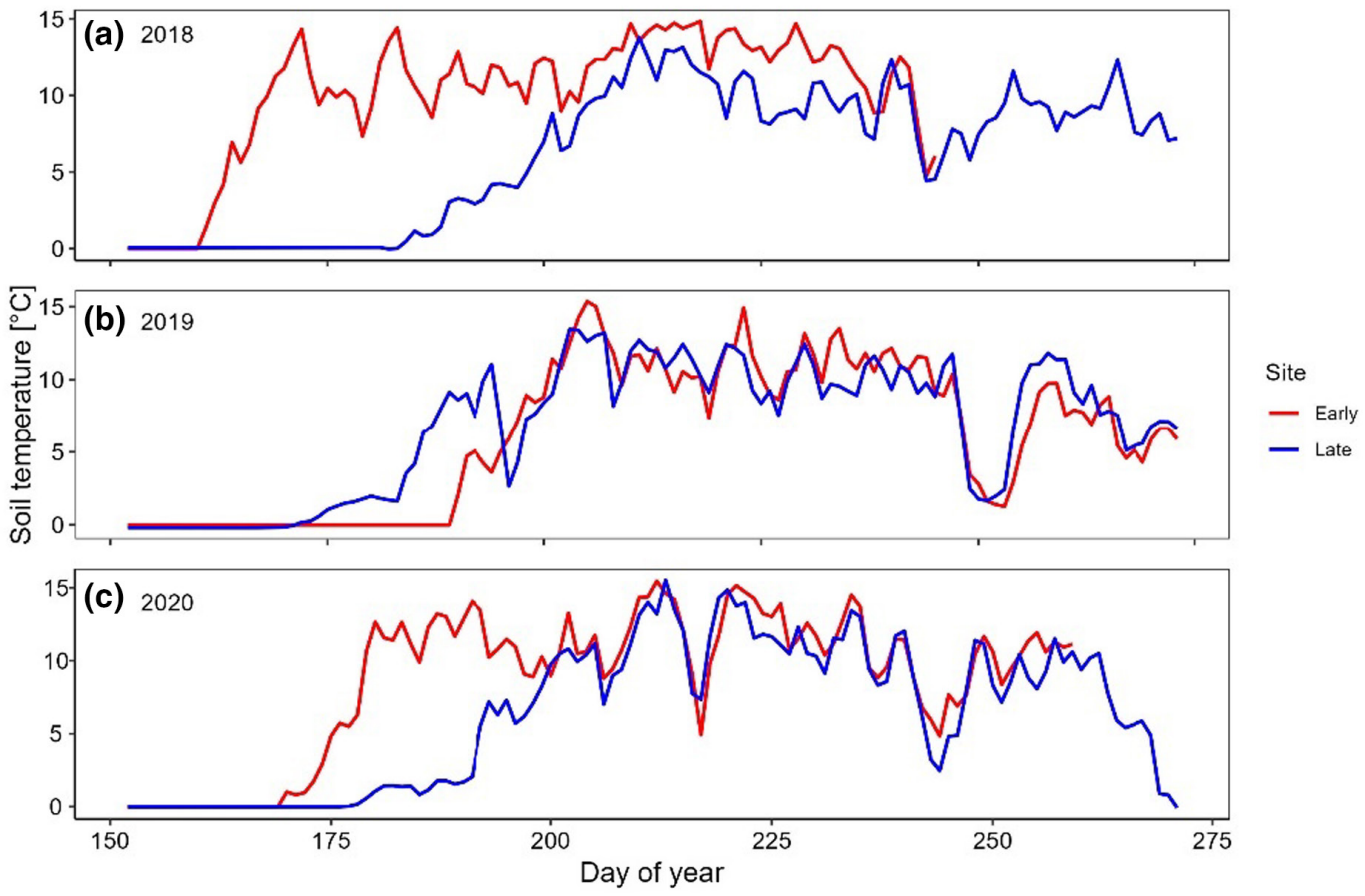
Daily mean soil temperatures at 2–3 cm depth (in°C) of both study areas starting from the 1st of June, for the years 2018 (a), 2019 (b) and 2020 (c). Time is expressed as days of year. Red = early snowmelt site (Mazia), Blue = late snowmelt site (Cimalegna).

The average soil temperature (Figure 2) was 0.6°C higher at the early snowmelt site. The most remarkable annual temperature contrast of 2.5°C occurred in 2018, favouring the early site. However, in 2019, temperatures were nearly identical at both locations, registering 8.8°C.

### 2.2 Phenological monitoring

To identify the differences in phenological development between the two study sites in response to these different snow regimes, we set up eight permanent plots in selected snowbeds at the late snowmelt site and four plots at the early snowmelt site, all of which contributed to vegetation patches belonging to the *Salicetum herbaceae* association. Each plot was a 16 m^2^ square divided into 16 subplots of 1 × 1 m. One plot at the early snowmelt site was reduced to 14 subplots because of its relatively smaller size. We installed a HOBO Pro v2 U23‐00x thermal probe (Onset Corp., Pocasset, MA, USA) in the centre of each snowbed. The probes measured hourly soil temperature data during the entire study period. We used this data to identify the day of snowmelt. Once the soil temperature was greater than 2°C, we considered that site to be snow‐free.

At the beginning of the first phenological season, we assessed the most abundant shared species at both study sites and selected five of them for monitoring: *Salix herbacea* L. and *Gnaphalium supinum* L. are typical snowbed species. *Poa alpina* L., a widespread alpine grass, at the late snowmelt site is partially replaced at the late snowmelt site by its pseudoviviparous subspecies *P*. *alpina* ssp. *vivipara* (Pierce et al., 2000). This subspecies exhibits compressed phenology and precocious bulbil germination, facilitating rapid clonal propagation within the abbreviated phenological season. *Veronica alpina* L. displays a wide ecological amplitude, thriving in both alpine meadow and snowbed communities. *Euphrasia minima* Schleich., one of the few annual alpine forbs, undergoes a compressed life cycle, rapidly transitioning from seed germination through flowering, seed set and senescence within a single short phenological season. Observations were performed approximately once every 2 weeks during the three monitored phenological seasons. The average observation frequency was higher at the beginning than at the end of the phenological season, consistent with other studies in alpine areas due to the crucial role of the onset of phenology in the life cycle of alpine plants (Carbognani et al., 2016; Filippa et al., 2015). Development stages and morphological characteristics were assigned and recorded after visual observation using a BBCH scale (Hack et al., 1992) adapted to snowbed plant communities as reference (Table 2). For each subplot, only the individual with the most advanced phenological stage was recorded. Subsequently, the median phenophase across all subplots was calculated for each plot and species, respectively. Representative shoots were coded and photographed at each phenological phase. A photographic handbook of phenophases was created during the first year of monitoring to reduce the potential survey bias (Quaglia et al., 2020). The adapted scale was used for the entire phenological cycle and was subdivided into six principal growth stages out of 10 on the general BBCH scale. Each principal growth stage was classified into secondary stages, describing points in time or shorter developmental intervals in the major growth stage, resulting in values between 0 (plants not visible) and 59 (completely senescent). In addition to the phenological monitoring, we applied a proximate sensing approach using phenocams installed in close proximity to one of the snowbeds at both sites.

**TABLE 2:** Adaptation of the BBCH scale (Hack et al., 1992) adapted for snowbed plant communities, representing the main phenological stages used during the surveys.

Phenophase		Description
		**Principal growth stage 0: Germination, sprouting and bud development**
09	A	Emergence: Cotyledons break through soil surface
	P	Bud shows green tips
	G	Emergence: Coleoptile breaks through soil surface
		**Principal growth stage 1: Leaf development**
10	A	Cotyledons completely unfolded
	P	First leaves separated
	G	First true leaf emerged from coleoptile
11	A,P,G	First true leaf, leaf pair or whorl unfolded
12	A,P,G	2 true leaves, leaf pairs or whorls unfolded
13	A,P,G	3 true leaves, leaf pairs or whorls unfolded
14–18		Stages continuous till (19)
19	A,P,G	More true leaves, leaf pairs, or whorls unfolded
		**Principal growth stage 2: Inflorescence emergence/heading**
20	A,P	Inflorescence or flower buds visible (soil level)
	G	Beginning of heading
23	A,P	Inflorescence or flower buds visible (beginning of elongation)
	G	Less than a half of inflorescence emerged
25	A,P	First individual flowers/flower styles visible (still closed)
	G	Half of inflorescence emerged (middle of heading)
29	A,P	First flower petals/flower visible (in petalled forms)
	G	Inflorescence fully emerged (end of heading)
		**Principal growth stage 3: Flowering (gamic/agamic reproduction for G)**
30	A,P	First flowers open (sporadically)
	G	First stamens/bulbils visible (sporadically), sheaf still closed
33	A,P	<50% of flowers open
	G	<50% of stamens/bulbils visible, sheaf begins to open
35	A,P	>50% of flowers open, first petals may be fallen
	G	>50% of stamens/bulbils visible, opened sheaf
37	A,P	Majority of petals dry or fallen
	G	Majority of stamens fallen/stem may begin to bend
39	A,P	End of flowering: fruit set visible
	G	End of flowering: fruit set visible/bent stem
		**Principal growth stage 4: development of fruit**
40	A,P	Fruit visible (unripe)
	G	Caryopsis watery ripe
43	A,P	<50% have reached the final size
	G	Early milk
45	A,P	>50% have reached the final size
	G	Milky ripe, medium milk
47	G	Late milk
49	A,P	Nearly all fruits have reached the final size
	G	Nearly all caryopses have reached the final size
		**Principal growth stage 5: Dissemination of fruit and seed (caryopsis/bulbils for G)**
50	A,P	Beginning of fruit abscission
	G	Beginning of dissemination (first glumes may be dry and empty)
53	A,P	<50% of seed dissemination
	G	<50% of caryopsis/bulbils disseminated
55	A,P	>50% of seed dissemination
	G	>50% of caryopsis/bulbils disseminated
59	A,P	Full dissemination
	G	Full dissemination (glumes totally dry and empty)

*Note*: The description of stages is given separately for annual (A), perennial (P) and graminoid (G) species.

### 2.3 Phenocam data

**FIGURE 3:**
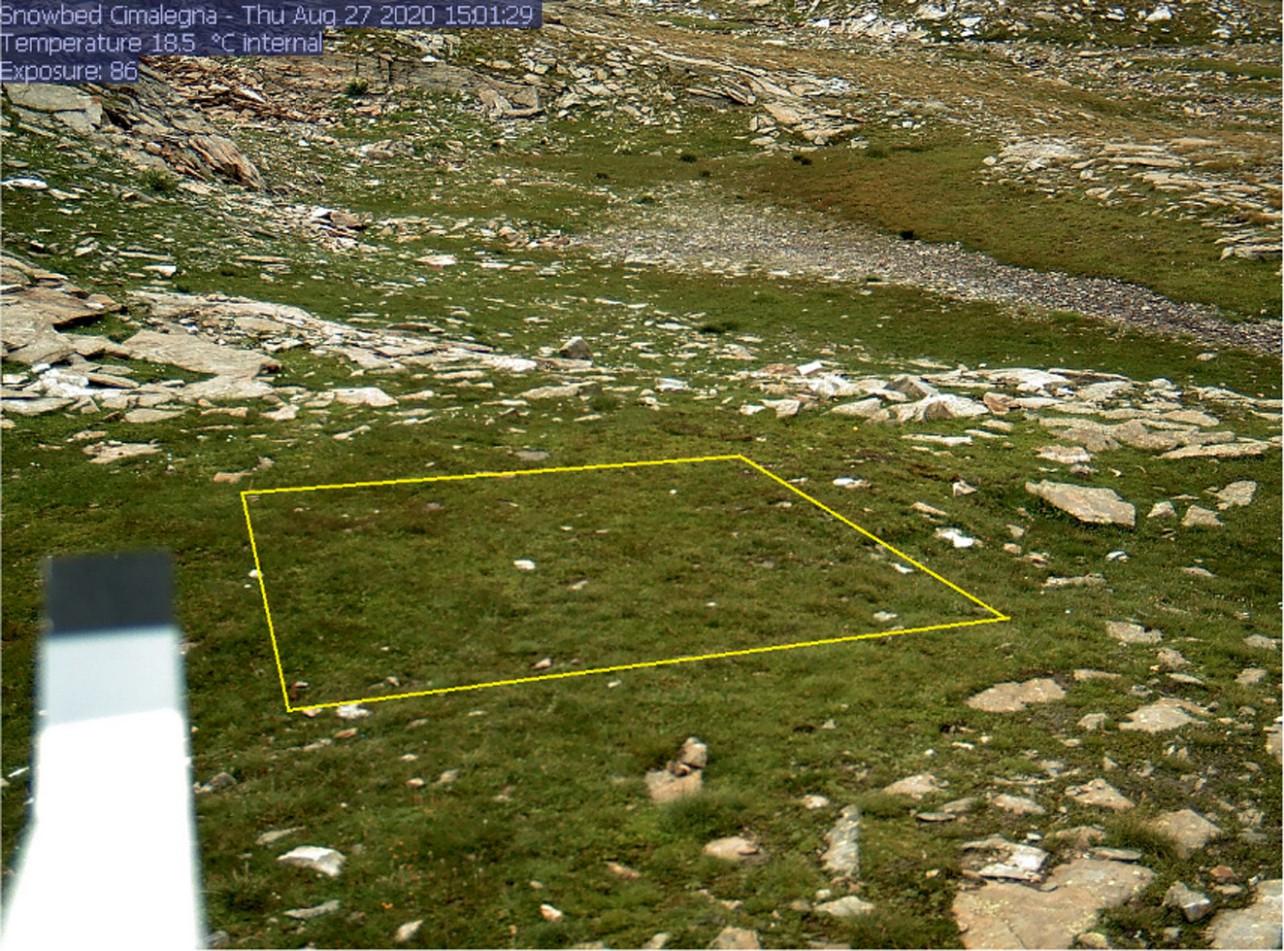
Phenocam image taken on July 27, 2020, at the Late Snowmelt. The yellow square highlights the Regions of Interest (ROI) for GCC data extraction.

At both study sites, Hybrid IP StarDot 5 Megapixel Netcams (StarDot Technologies, Buena Park, CA, USA) were installed approximately 2 m high, facing the snowbeds at similar viewing angles. Images were captured automatically every hour during daylight periods over the entire phenological season at both study sites. For the late snowmelt site, we defined a region of interest (ROI) of 4 m × 4 m (Figure 3), whereas we defined two smaller ROIs at the early snowmelt site because the snowbed was smaller and irregularly shaped. ROIs were in the centre of the images to minimise the front‐rear vegetation weighting effects. For both sites, masks were created, from which the colour information could be extracted from an RGB image.

The mean pixel value for each image was determined across the ROI mask for the RGB colour channels. The green chromatic coordinate (GCC) index is calculated using the following equation:

$$\mathrm{GCC}=\frac{G}{\left(R+G+B\right)}$$

where *G* represents the green, *R* represents the red and *B* represents blue bands (Nijland et al., 2014; Reid et al., 2016; Richardson et al., 2015; Sonnentag et al., 2012). The GCC was then employed as a parameter in generalised additive mixed models (GAMM).

Because there are many sources of error and a large number of images from repeated digital imagery, we extracted the values from the images using a range of methods aimed at ensuring data quality. We filtered for image errors, such as precipitation or fog; excluded images with exposure times longer than 50 s, exposure ratios lower than 2 and acquisition times earlier than 10 AM and later than 5 PM with long exposure times; and applied clustering and brightness filters. Furthermore, we calculated the 90th percentile value of the GCC across the remaining images over a four‐day moving window. This combination of methods has been shown to be effective in minimising variations due to weather and illumination geometry (Sonnentag et al., 2012). More detailed information about the imaging and data processing routines performed can be found in Richardson et al. (2018) and Rossi et al. (2019).

### 2.4 Data preparation and statistical analysis

We related the phenological stages observed in the field to the season‐ and climate‐related variables day of year (DOY), used as a proxy for photoperiod; days from snowmelt (DFSM), to include the effect of snow dynamics; and growing degree days (GDDs), computed as a thermal sum (Oberbauer et al., 2013), to identify the most meaningful predictor. GDD was calculated for every observation date and plot during the snow‐free period (Figure 1). Our study employed GDD using a base temperature of 0°C to capture also the growth responses of snowbed species to low temperatures (Carbognani et al., 2016; Körner, 2021; Legault & Cusa, 2015), although temperature‐growth relationships are strongly non‐linear, with only little growth below 5°C (Nagelmüller et al., 2015).

We employed GAMMs to investigate the role of climatic variables in driving plant phenological development using the R package ‘gamm4’ (Wood & Scheipl, 2020). GAMMs combine the flexibility of generalised additive models (GAMs) in modelling non‐linear relationships with the ability of linear mixed models to account for nested data structures (Zuur et al., 2009). This approach is well‐suited for ecological studies, as it can handle complex effects of temperature and snowmelt patterns on phenology while appropriately accounting for the hierarchical nature of the data.

For each species, we constructed separate GAMMs using the ordinal BBCH scale values as a quasi‐continuous response variable representing phenological progression. Median phenophase values across the three monitoring years were modelled as smooth functions of day of year (DOY), DFSM, GDDs and their combinations using penalised regression splines as fixed effects. To select the predictor variables, we utilised the Akaike information criterion (AIC) (Akaike, 1974) to assess model fit and complexity. The model was evaluated by analysing the standardised residuals, fitted values and errors. We screened all fixed‐effects predictor variables for correlations and found that they were highly correlated (Pearson's *r* > .90). Therefore, for further analysis and prediction, we used the model best fitted to a single variable.

To quantify the magnitude of species' responses, we calculated the phenological development rates (PDRs) for each species, year and site. PDR was derived as the slope factor of the regression line between the first relevant phenological observation (phenophase ≥09) and the final observation prior to phenological cycle completion (phenophase <59). We then calculated the 3‐year average for each species and plotted it to assess potential long‐term adaptations or plasticity in response to advancing snowmelt and changing snow dynamics. To evaluate interspecific variations and ecosystem phenology, we partitioned the phenological season based on the GCC derived from phenocam imagery into five primary phenological events: start of the season (SOS), start of the peak of the season (SPS), peak of the season (POS), end of the peak of the season (EPS) and end of the season (EOS). A corresponding DOY was assigned to each stage. The SPS and EPS were determined as the periods during which the GCC fell within 95% of the maximum GCC value and these calculations were conducted separately for each site.

Finally, we used data from both study sites to assess whether we could predict phenological developments for a future with earlier snowmelt and longer vegetation periods using GAMM and the predicted function. We determined the median phenophase for each species and plot using subplot data. These median phenophase values were then modelled with GDDs as the fixed effect and ‘Plot’ and ‘Year’ as random effects. This analysis utilised data from late snowmelt sites to evaluate their potential in predicting phenological changes under varying climatic conditions. GAMMs fitted with data from the late snowmelt site were used to predict phenological progression at the early snowmelt site and the predictions were validated against observed monitoring data. In the case of adaptation to different snowmelt timings, we would expect differences in phenological development when compared to GDD. All statistical analyses were conducted in R 4.2.2 (R Core Team, 2022).

## 3 RESULTS

### 3.1 Microclimatic drivers

At the late snowmelt site, GAMMs revealed strong correlations between species phenophases and DOY, DFSM and GDD for all plots, as these variables are all time‐dependent (Table 3). However, *R* ^2^ values were consistently lower for DOY models compared to DFSM and GDD models. DOY models also exhibited the highest variability in *R* ^2^ across species, plots and years. GDD models had slightly higher average *R* ^2^ values than DFSM models and lower AIC values, indicating better fit. Including additional covariables in the GAMMs did not improve *R* ^2^ compared to GDD‐only models.

**TABLE 3:** *R* ^2^ and AIC (Akaike information criterion) values of the GAMMs fitted with the phenophases of the study species at the late and early snowmelt site and DOY, DFSM and GDDs.

Species	Site	GDD		DOY		DFSM	
		*R* ^2^	AIC	*R* ^2^	AIC	*R* ^2^	AIC
*Salix herbacea*	Late	.929	1048.18	.838	1127.92	.895	1090.66
	Early	.969	529.99	.697	755.08	.967	540.09
*Poa alpina*	Late	.941	1015.20	.863	1035.90	.903	1053.38
	Early	.937	487.60	.815	555.50	.936	485.62
*Veronica alpina*	Late	.923	1068.49	.894	1072.66	.896	1092.77
	Early	.958	524.98	.782	612.79	.959	530.92
*Gnaphalium supinum*	Late	.905	1112.67	.857	1111.34	.881	1126.47
	Early	.968	524.45	.789	612.79	.965	534.40
*Euphrasia minima*	Late	.950	999.84	.887	1012.64	.924	1040.86
	Early	.869	664.00	.830	672.00	.871	671.34
Average	Late	.930	1048.88	.868	1072.09	.900	1080.83
	Early	.940	546.20	.783	641.63	.940	552.47

### 3.2 Interspecific variability and the PDR

**FIGURE 4:**
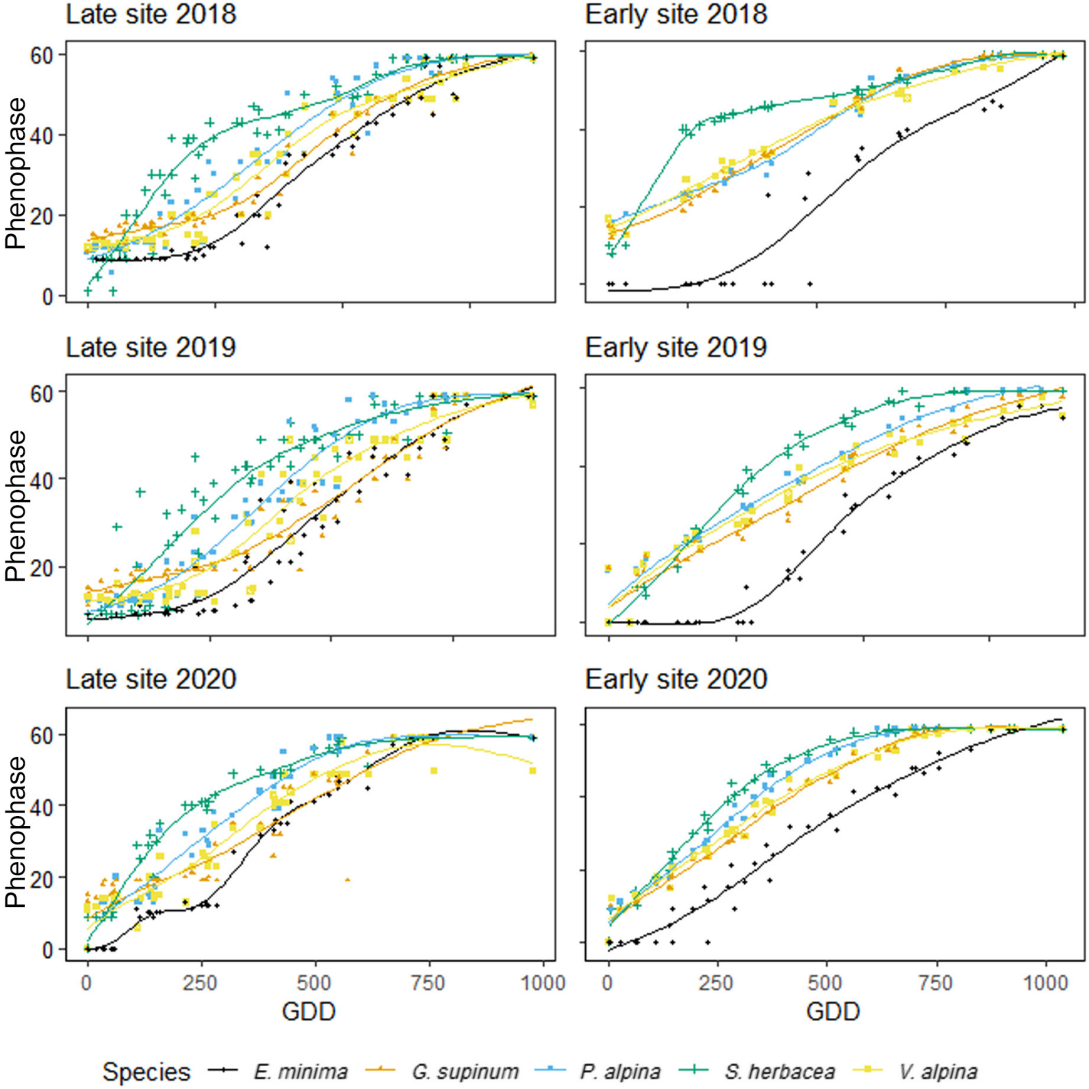
Relationships between median phenophases of all 5 monitored plant species and the GDDs using the species phenophase value and GDD for the late and early snowmelt site.

We observed high variability in phenological development among species and years, albeit with some consistent patterns (Figure 4). *S*. *herbacea*, generally had the earliest phenophase transitions, rapidly progressing to senescence (phenophase 50–59) before other species. In contrast, the annual *E*. *minima* displayed a delay of up to 250 GDDs relative to other species at most plots. *V*. *alpina*, *P*. *alpina* and *G*. *supinum* exhibited intermediate phenological progressions. Despite delayed leaf unfolding, *E*. *minima* frequently attained fruit development (phenophase 40–50) concurrently with intermediate species.

**FIGURE 5:**
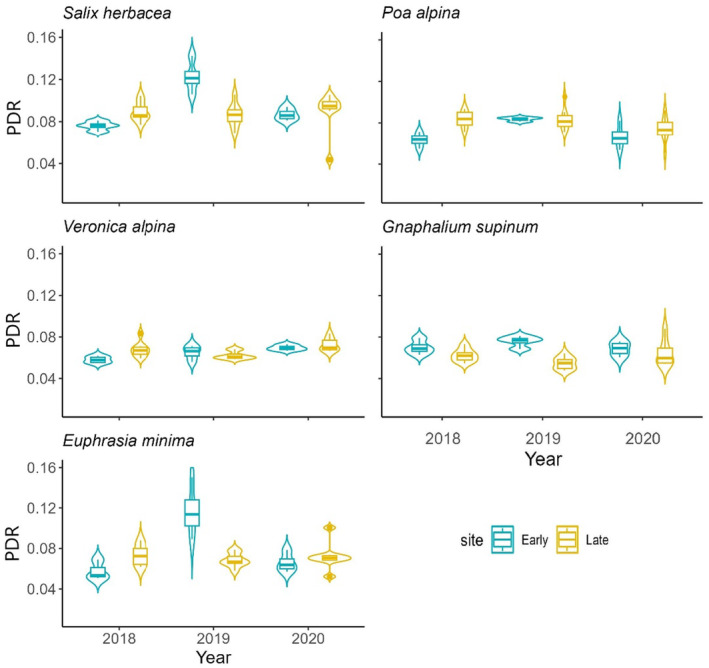
The PDR of each study in all 3 years at both study sites for the five monitored species. Blue boxes represent data from the ‘Early’ study site and yellow boxes represent data from the ‘Late’ snowmelt site.

Over the three‐year period, *S*. *herbacea* had the fastest PDR at both study sites, followed by *E*. *minima*, *P*. *alpina* and *G*. *supinum*. *V*. *alpina* exhibited the slowest development (Table 4). Species displayed varying PDRs between the two sites, with greater year‐to‐year variability than site differences (Figure 5). At the early snowmelt site in 2018, snowmelt occurred exceptionally early. Consequently, *P*. *alpina* and *V*. *alpina* showed a faster phenological development compared to the late snowmelt site (Table S1). Conversely, in 2019, when snowmelt was considerably delayed at the early site, *S*. *herbacea*, *G*. *supinum* and *E*. *mini*ma showed faster development at this site, with substantially higher PDRs compared to the late snowmelt site (Figure 5, Table S1).

**TABLE 4:** Statistical evaluation of the difference in the PDR between the early and the late snowmelt sites.

Species	Site	*p*	*M*	*SD*
*Salix herbacea*	Late	.36	0.089	0.01
	Early		0.095	0.005
Poa alpina*	Late	.013*	0.08	0.004
	Early		0.069	0.008
*Veronica alpina*	Late	.18	0.068	0.004
	Early		0.064	0.003
*Gnaphalium supinum*	Late	<.001***	0.06	0.006
	Early		0.072	0.005
*Euphrasia minima*	Late	.3	0.071	0.009
	Early		0.082	0.015

*Note*: Values were calculated as average values of every plot and all 3 years. *p*, likelihood of observed results by chance under the null hypothesis; M, mean PDR; SD, standard deviation of these values. Significance levels: *p < .05, ***p < .001.

### 3.3 Mean ecosystem phenology

**FIGURE 6:**
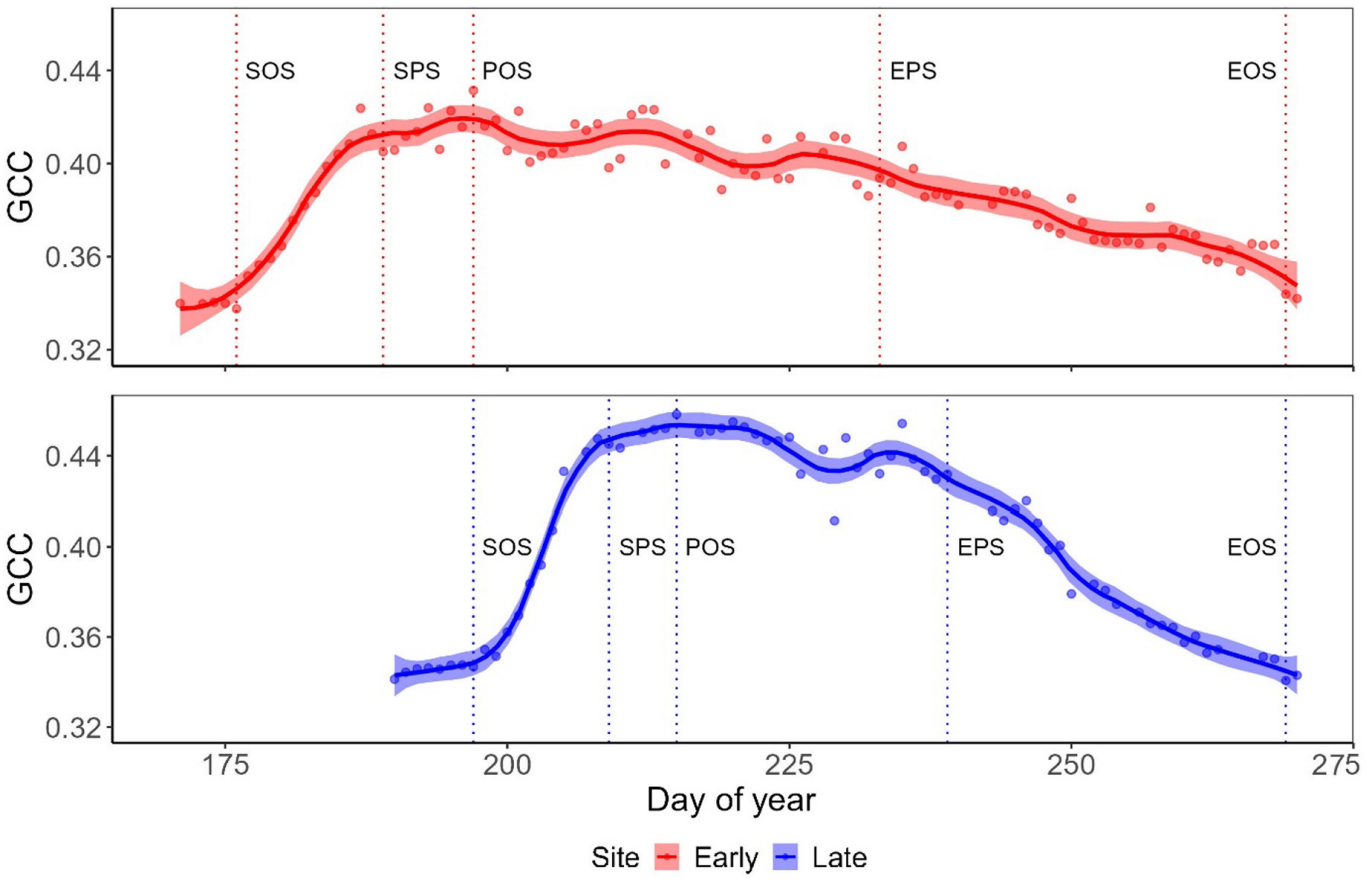
Daily maximum values for the Green Chromatic Coordinate (GCC) at the early (red) and late (blue) snowmelt sites for the phenological season 2020. Main phenological events SOS (start of season), POS (peak of season) and EOS (end of season) are indicated for both sites.

Mean ecosystem phenology, (Figure 6) showed a rapid rise in GCC values after snowmelt, followed by a plateau during the primary phenological season (SPS – EPS) and a gradual decline towards the end of the season. SOS at the early snowmelt site occurred on DOY 176, whereas at the late snowmelt site, the phenological season started 21 days later, on DOY 197. POS at the early site was reached on DOY 197, 21 days after SOS, with GCC values over 0.42 (max. 0.44), whereas it occurred on DOY 215 at the late snowmelt site after only 18 days with a maximum GCC value of 0.46. Akin to other alpine sites, the phenological season at both study locations concluded with vegetation browning (Figure 6) in late September (Körner et al., 2022). In total, the phenological season lasted approximately 3 months (mid‐June to late September) at the early snowmelt site and 2 months (mid‐July to late September) at the late snowmelt site. The period from SPS to EPS accounted for slightly less than half of the total phenological season at both sites. Notably, the GCC values at the late snowmelt site averaged 10% higher than those at the early snowmelt site, featuring a more rapid initial increase and steeper decline towards the end of the phenological season.

When comparing the phenological stages of the individual study species to the GCC, the model demonstrated strong performance, attaining an adjusted *R* ^2^ of .901, accounting for 94.9% of the deviance. Notably, *S*. *herbacea* and *P*. *alpina* contributed most in explaining the variations in GCC values (Table 5).

**TABLE 5:** Results of the GAMM fitted with the Green Chromatic Coordinate (GCC) as dependent variables and the species phenophases as independent variables to investigate the importance and contribution of each species to the ecosystem greenness.

Parameter	Estimate	Std. Error	*t*‐Value	Pr(>|*t*|)
(Intercept)*	0.394692	0.002643	149.3	<0.001***
Smooth term	edf	Ref.df	*F*	*p*‐Value
s(*E*. *minima*)	2.13	2.51	0.78	.348
s(*S*. *herbacea*)*	1	1	15.84	.004**
s(*G*. *supinum*)	1	1	3.21	.111
s(*V*. *alpina*)	1	1	0.19	.674
s(*P*. *alpina*)*	2.73	2.94	6.29	.015*

*Note*: *N* = 17.

Significance levels: *p < .05, **p < .01, and ***p < .001.

Moreover, when examined individually, the early species *S*. *herbacea* exhibited the most robust positive correlation with the GCC among all species (*R* ^2^ = .37), followed by *P*. *alpina* (*R* ^2^ = .23). In contrast, *E*. *minima*, the species with the latest phenology, displayed the only negative correlation with GCC (*R* ^2^ = −.31). *V*. *alpina* (*R* ^2^ = −.04) and *G*. *supinum* (*R* ^2^ = −.02) showed no discernible correlations. While the phenological development of *S*. *herbacea* was positively correlated and that of *E*. *minima* was negatively correlated with the GCC at the early and late snowmelt sites, the results for the other species varied between sites. *P*. *alpina* exhibited a positive correlation at the early snowmelt site but showed no correlation at the late snowmelt site. *V*. *alpina* and *G*. *supinum* were negatively correlated at the late snowmelt site and positively correlated at the early snowmelt site (Figure S2).

### 3.4 Predictive modelling

**FIGURE 7:**
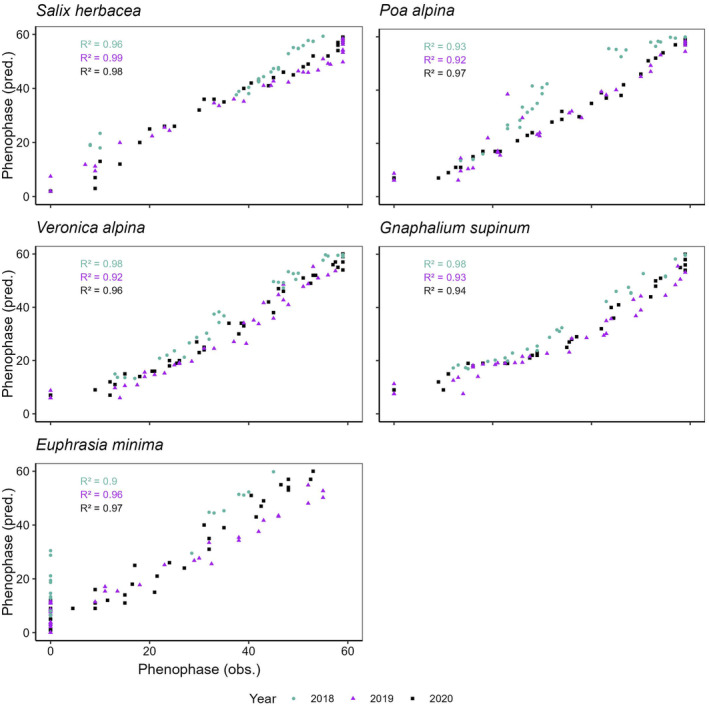
Comparison of predicted vs measured phenological phases based on the GAMM predictions (GDD) for the 5 study species. The *y*‐axis represents predicted (pred.) values and the *x*‐axis represents observed (obs.) values.

The phenological stages predicted for the early snowmelt site, using the data collected at the late snowmelt site, demonstrated a strong correlation with the measured values at the early site (Pearson's correlation *r* > .95, Table S3). However, when comparing the predictions to in‐situ observations at early snowmelt sites, considerable differences emerged (Figure 7). Overall, the models performed better for *S*. *herbacea* and *V*. *alpina* but showed less accurate predictions for *G*. *supinum* and *E*. *minima*. Furthermore, prediction accuracy varied across phenological stages depending on species and year. For example, the models overestimated the development of *E*. *minima* after flowering (BBCH stage 30) in 2018 and 2020 but underestimated the phenological development of *G*. *supinum* in 2019 and 2020 after stage 20. These substantial disparities between predicted and observed values may partly stem from potential systematic errors. The most accurate phenophase predictions were for *S*. *herbacea* and *V*. *alpina*, with average errors of 2.8 and 3.1 phenophases according to the BBCH scale, followed by *P*. *alpina* (±3.6 stages) and *G*. *supinum* (4 stages), while the average error for *E*. *minima* was 6.6 phenophases.

## 4 DISCUSSION

In our study, timing of snowmelt, a crucial factor initiating the alpine growing season (Inouye & Wielgolaski, 2013), varied between years and sites. However, post‐melting soil temperatures at both study sites were similar (Figure 1), aligning with observations from other Alpine regions (Körner et al., 2022). While the temperature, photoperiod and soil moisture also have the potential to impact phenology (Hülber et al., 2006; Keller & Körner, 2003; Kudo & Suzuki, 1999), we found that the timing of snowmelt and temperature were the main drivers, as species opportunistically tracked snowmelt timing. Species‐specific differences at a given site can be partially explained by the degree of flower preformation in the previous year and species‐specific (genetic) developmental programs. However, by comparing early and late snowmelt sites, we can draw conclusions regarding their responsiveness to changes, as the influence of environmental factors can vary among species within the same habitat, contributing to the observed species‐specific responses (Keller & Körner, 2003).

### 4.1 Interspecific differences

When correlating the species' phenological development to the GDD, irrespective of the timing of snowmelt onset, each species showed a specific timing. The snowbed specialists *S*. *herbacea* and *G*. *supinum* responded directly to snowmelt, demonstrating their highly opportunistic nature and adaptation to short phenological seasons (Petraglia et al., 2014; Sedlacek et al., 2015).

*S*. *herbacea*, a prostrate shrub, initiates leaf growth and photosynthesis very early in the phenological season, sometimes even while still covered by snow (Cortés et al., 2014; Little et al., 2016). However, the apparent early onset in *S*. *herbacea* can partially be attributed to its above‐ground bud break, which is more easily observable compared to the other perennial species with apical meristems below ground. Despite the potential risks of freezing damage, reduced leaf size (Kudo et al., 2001), hampered reproduction (Lluent et al., 2013; Wheeler et al., 2016) and nutrient availability issues (Carbognani et al., 2016; Little et al., 2016) under earlier snowmelt scenarios, early phenology species like *S*. *herbacea* and *G*. *supinu*m respond more strongly to advancing snowmelt (Petraglia et al., 2014). *G*. *supinum*, a rosette herb, starts flushing 1–2 cm below ground and unfolds its leaves and flower buds based on specific thermal sums or daylengths, minimising the risk of freezing damage (Keller & Körner, 2003). While both species profit from earlier snowmelt, their different morphotypes and strategies for leaf opening timing make it challenging to identify the physical start of leafing with equal confidence.

*E*. *minima*, as annual species, consistently reached phenological stages last and did not advance its phenology with earlier snowmelt, only prolonging its prefloration period (Petraglia et al., 2014). However, it might benefit from a longer phenological season. Its annual life cycle causes a phenological delay during early growth stages due to seed dormancy breaking and germination (Liebst & Schneller, 2008). *E*. *minima* compensates through a compressed phenological sequence, shorter vegetative phase and profuse seed production (Brown et al., 2020). This results in a higher PDR and a ‘catch up’ with perennial counterparts. In short phenological seasons, like 2019, *E*. *minima* was the only species still partially blooming during the first snowfall. It mitigates such events by spreading germination over 3 years, suggesting a persistent seed bank formation (Liebst & Schneller, 2008). *P*. *alpina* and *V*. *alpina* showed intermediate phenological patterns, mostly unaffected by year‐to‐year snowmelt timing variations at both study sites.

### 4.2 Site specific differences and interannual variability

Further comparative analyses revealed site‐specific differences in PDR between the early and late snowmelt sites only for *G*. *supinum* and *P*. *alpina*. *P*. *alpina* exhibited a higher PDR at the late snowmelt site, while *G*. *supinum* displayed a higher PDR at the early site. This suggests that the snowbed species *G*. *supinum* might, next to *S*. *herbacea*, be the most opportunistic species in this survey (Körner, 2021), by initialising phenological development even at low temperatures. Conversely, the faster PDR of *P*. *alpina* at the late snowmelt site suggests it might not be able to capitalise on earlier snowmelt, as previous results have shown, possibly due to temperatures remaining low at the beginning of the phenological season (Nagelmüller et al., 2015; Petraglia et al., 2014). Differences between sites for *P*. *alpina* may partly also stem from the presence of *P*. *alpina* ssp. *vivipara* at the late snowmelt site. *P*. *alpina* ssp. *alpina* reproduces sexually through seeds, with a growth cycle including flowering, seed maturation and dispersal. In contrast, *P*. *alpina* ssp. *vivipara* reproduces asexually through bulbils that develop directly on the inflorescence. (Liebst & Schneller, 2008; Pierce et al., 2000). Nonetheless, changes in PDR at the early snowmelt site in response to varying snowmelt timing may indicate a sensitivity to snowmelt timing (Kudo, 2020) for *P*. *alpina*.

*S*. *herbacea* and *E*. *minima* showed no discernible differences over the three‐year period but displayed pronounced interannual variability, likely due to their responses to changes in temperature accumulation at the early snowmelt site. Both species maintained similar PDR at the late snowmelt site but exhibited remarkable differences at the early snowmelt site. In 2018, under very early snowmelt conditions, both species had slower phenological growth compared to an ‘average’ year (2020), while in 2019, marked by a considerably delayed snowmelt at the early site, a notably higher PDR was observed. These variations in PDR between sites may be partially attributed to the non‐linear plant growth responses to temperature, with most growth occurring above 5°C but stagnating above 10°C (Nagelmüller et al., 2015). The pronounced year‐to‐year variations underscore the role of snowmelt in driving the phenological cycle of alpine plant species (Hülber et al., 2010; Winkler et al., 2018; Wipf et al., 2009). These variations result from evolutionary adaptations that ensure vital processes such as nutrient recovery, timely senescence and fruit maturation, becoming more pronounced in years with notably early or late snowmelt (Körner & Hiltbrunner, 2021). Snowbed specialists have evolved an opportunistic phenology, often facilitated by clonal propagation (de Witte et al., 2012), to maximise resource acquisition and reproductive success within the narrow phenological season imposed by temperature and snowmelt patterns. This strategy confers resilience to competition from migrating species. However, the dual impact of reduced snow cover protection and increased competition from invading grassland plants could threaten these specialist species due to climate warming (Hülber et al., 2011). Although the synchronisation ensures survival and fitness, it also limits the advantages they can derive from a warmer climate (Körner & Hiltbrunner, 2021).

The three‐year analysis revealed only marginal differences in PDRs between early and late snowmelt sites for four of the five studied species, highlighting their resilience and ability to capitalise on temperature and snowmelt timing variations. While our results suggest a good adaptability of these species to changing conditions, other findings indicate that a conservative phenological approach may not be sufficient to mitigate the adverse effects of climate change (Notarnicola et al., 2023; Rixen et al., 2010). It remains uncertain whether the opportunistic phenology is enough to avoid changes in species composition. However, it is evident that snowbed species exhibit a high degree of phenological plasticity, at least during the early phenological season, enabling them to track and respond to variations in temperature and snowmelt patterns. Furthermore, the greater year‐to‐year variations compared to the differences between early and late snowmelt sites may be explained by the fact that arctic and high alpine plants often respond more strongly to short‐term climatic changes than to changes over time (Bjorkman et al., 2020). This may, in part, indicate regional and/or long‐term adaptations to evolving temperature and snowmelt patterns (Weißhuhn et al., 2012).

### 4.3 Optical vegetation index

The green chromatic coordinate (GCC) patterns observed at early and late snowmelt sites revealed distinct trends, similar to the differences found alpine grassland (Julitta et al., 2014) and in arid and temperate grasslands (Browning et al., 2017; Watson et al., 2019). At both study sites a rapid GCC increase was measured after snowmelt for approximately 3 weeks, followed by an early peak resembling the lower elevation late spring ‘green spike’ (Keenan et al., 2014). Subsequently, trends diverged, with the late site showing a notably shorter plateau phase analogous to the gradual summer green‐down (Richardson et al., 2018) and a more rapid autumn green‐down exclusive to the late site. In contrast, the early site displayed a gradual decline attributed to its extended season (Linderholm, 2006; Menzel & Fabian, 1999). While phenocams effectively capture general ecosystem phenological patterns, representing individual species' phenology remains challenging due to variations in species composition, abundance and phenological timing (Julitta et al., 2014; Keenan et al., 2014). Furthermore, varying light conditions, small plant structures, occlusion by vegetative parts and high biodiversity can influence results and potentially contribute to observed differences between sites (Gallmann et al., 2022; Mann et al., 2022). Nevertheless, species‐level analyses are important, as climate sensitivity varies significantly among organisms within the same taxonomic and trophic groups (Thackeray et al., 2016).

### 4.4 Predictive modelling

The predictive quality of the models varied between years and phenological stages. Predictive quality may be influenced by snowmelt timing due to challenges in integrating species‐specific responses of opportunistic behaviour to snowmelt variations. This observation suggests a broader trend of diminishing phenological responses to climate‐related changes throughout the phenological season, aligning with documented trends in lowland species (Hülber et al., 2010). Predictions were generally more accurate for some species (*S*. *herbacea* and *V*. *alpina*) and less accurate for others (*P*. *alpina* and *G*. *supinum*), irrespective of their life strategy and level of phenological opportunism. These results underscore the importance of considering the interplay among snowmelt timing, temperature and phenology, as species‐specific responses challenge predictive model accuracy, emphasising caution when extrapolating results to future climate scenarios.

## 5 CONCLUSION

Our results highlight that snowbed species exhibit highly opportunistic responses to advancing snowmelt by tracking changes in snowmelt timing while maintaining species‐specific developmental patterns. Year‐to‐year variation influenced phenology more than differences in local snowmelt dates, suggesting these species' potential to adapt to future earlier melting dates. Despite phenological plasticity, species from surrounding grasslands may benefit from extended growing seasons, whereas snowbed species may be outcompeted due to non‐opportunistic senescence driven by internal clocks. Therefore, it is essential to focus not only on species but also on ecosystem levels, where phenocams have proven valuable despite limitations in explaining species‐level variance. This underscores the necessity of comprehensive approaches that integrate multiple monitoring methods throughout the entire phenological season for understanding alpine phenological responses to global warming.

## AUTHOR CONTRIBUTIONS

**Harald Crepaz:** Conceptualization (equal); data curation (lead); formal analysis (lead); investigation (equal); methodology (equal); project administration (equal); visualization (lead); writing – original draft (lead); writing – review and editing (lead). **Elena Quaglia:** Conceptualization (equal); data curation (equal); formal analysis (supporting); methodology (equal); writing – review and editing (supporting). **Giampiero Lombardi:** Conceptualization (equal); methodology (equal); project administration (equal); writing – review and editing (equal). **Michele Lonati:** Conceptualization (equal); methodology (equal); project administration (equal); writing – review and editing (equal). **Simone Ravetto Enri:** Conceptualization (equal); data curation (equal); investigation (equal); methodology (equal); writing – review and editing (equal). **Mattia Rossi:** Data curation (equal); investigation (equal); methodology (equal); software (equal); writing – review and editing (equal). **Stefan Dullinger:** Supervision (equal); writing – review and editing (equal). **Ulrike Tappeiner:** Project administration (equal); supervision (equal); writing – review and editing (equal). **Georg Niedrist:** Conceptualization (equal); investigation (equal); methodology (equal); project administration (equal); supervision (equal); writing – review and editing (equal).

## CONFLICT OF INTEREST STATEMENT

The authors declare no conflicts of interest related to this study.

## Supporting information


Appendix S1.

## Data Availability

The datasets generated and analysed during the current study are available on Zenodo at https://doi.org/10.5281/zenodo.10691482.
