# Supplementary material for: Phenological responses of alpine snowbed communities to advancing snowmelt
Source: Ecol Evol. 2024 Jul 14;14(7):e11714. doi: 10.1002/ece3.11714 (PMC11246788; doi:10.1002/ece3.11714)
Supplement: Supplementary file 1 — Appendix S1. [file ECE3-14-e11714-s001.docx]

**Supplementary material**

**S1.** Statistical evaluation of the differences in phenological growth rate (PGR) between early and late snowmelt sites. Values were calculated as the average for each plot for each year and species. T, Student’s t-value; d.f., degrees of freedom; p, probability of test statistic t under the null hypothesis; M, mean cover (%) or NDVI-value; SD, standard deviation of these values.

| **Species** | **Year** | **Site** | ***df*** | ***t*** | ***p*** | ***M*** | ***SD*** |
| --- | --- | --- | --- | --- | --- | --- | --- |
| ***Salix herbacea*** | **2018*** | Early | 10 | 3.414 | 0.007 | 0.0761 | 0.0044 |
|  |  | Late |  |  |  | 0.0893 | 0.0090 |
|  | **2019*** | Early | 5 | -4.306 | 0.008 | 0.1227 | 0.0150 |
|  |  | Late |  |  |  | 0.0863 | 0.0111 |
|  | **2020** | Early | 9 | 0.445 | 0.667 | 0.0868 | 0.0057 |
|  |  | Late |  |  |  | 0.0901 | 0.0194 |
| ***Poa alpina*** | **2018*** | Early | 7 | 4.486 | 0.003 | 0.0633 | 0.0070 |
|  |  | Late |  |  |  | 0.0835 | 0.0079 |
|  | **2019** | Early | 8 | -0.030 | 0.977 | 0.0836 | 0.0021 |
|  |  | Late |  |  |  | 0.0835 | 0.0109 |
|  | **2020** | Early | 6 | 0.977 | 0.366 | 0.0663 | 0.0120 |
|  |  | Late |  |  |  | 0.0736 | 0.0124 |
| ***Veronica alpina*** | **2018*** | Early | 10 | 3.167 | 0.010 | 0.0582 | 0.0033 |
|  |  | Late |  |  |  | 0.0680 | 0.0074 |
|  | **2019** | Early | 4 | -0.785 | 0.477 | 0.0651 | 0.0068 |
|  |  | Late |  |  |  | 0.0622 | 0.0038 |
|  | **2020** | Early | 10 | 1.099 | 0.298 | 0.0696 | 0.0027 |
|  |  | Late |  |  |  | 0.0724 | 0.0060 |
| ***Gnaphalium supinum*** | **2018** | Early | 6 | -1.890 | 0.108 | 0.0701 | 0.0068 |
|  |  | Late |  |  |  | 0.0624 | 0.0062 |
|  | **2019*** | Early | 7 | -6.240 | <0.001 | 0.0759 | 0.0053 |
|  |  | Late |  |  |  | 0.0547 | 0.0060 |
|  | **2020** | Early | 10 | -1.208 | 0.255 | 0.0687 | 0.0070 |
|  |  | Late |  |  |  | 0.0610 | 0.0151 |
| ***Euphrasia minima*** | **2018** | Early | 4 | 2.344 | 0.079 | 0.0576 | 0.0098 |
|  |  | Late |  |  |  | 0.0734 | 0.0103 |
|  | **2019*** | Early | 3 | -3.668 | 0.035 | 0.1167 | 0.0259 |
|  |  | Late |  |  |  | 0.0685 | 0.0068 |
|  | **2020** | Early | 8 | 0.949 | 0.371 | 0.0658 | 0.0094 |
|  |  | Late |  |  |  | 0.0721 | 0.0133 |


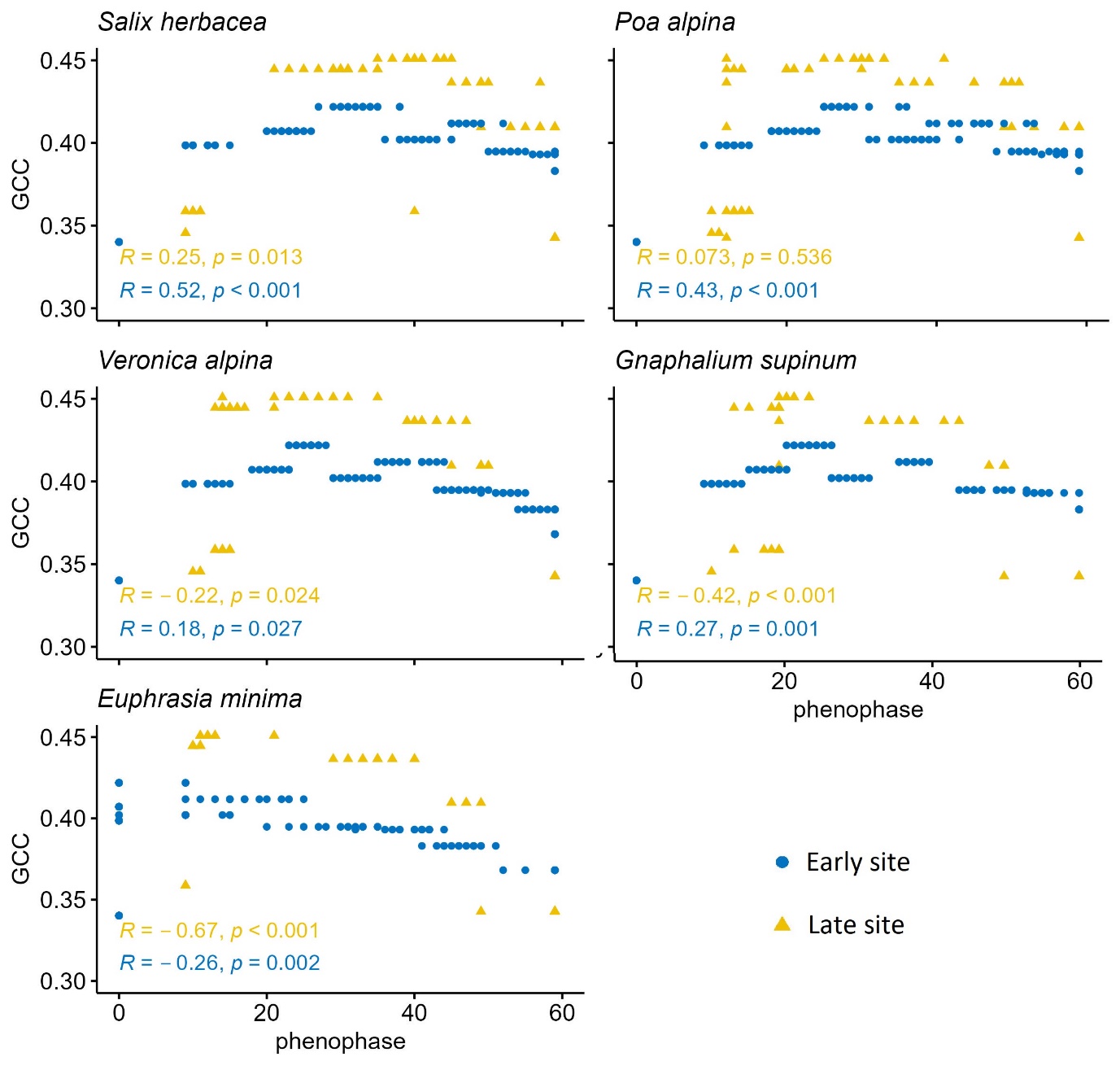


**S2.** Correlations between Green Chromatic Coordinate (GCC) and phenophases of five study species at early (blue dots) and late (yellow triangles) snowmelt sites. Phenophases are denoted by progressive numbers following the BBCH scale adaptation (Hack et al., 1992), with Pearson's correlation coefficient (R) and significance indicated in each chart.

**S3.** Correlations between the phenological development of the snowbed species predicted by the predictive models, fitted with data from the late snowmelt site, and the *in-situ* monitored values at the early snowmelt site.

| *Species* | *Plot 1* | *Plot 2* | *Plot 3* | *Plot 4* | *Early site* |
| --- | --- | --- | --- | --- | --- |
| *Salix herbacea* | 0.998 | 0.988 | 0.987 | 0.995 | 0.992 |
| *Poa alpina* | 0.981 | 0.995 | 0.996 | 0.984 | 0.989 |
| *Veronica alpina* | 0.979 | 0.993 | 0.997 | 0.979 | 0.987 |
| *Gnaphalium supinum* | 0.972 | 0.973 | 0.972 | 0.972 | 0.972 |
| *Euphrasia minima* | 0.988 | 0.991 | 0.994 | 0.983 | 0.989 |
